# Ontogenesis of the Mouse Ocular Surface Lymphatic Vascular Network

**DOI:** 10.1167/iovs.64.15.7

**Published:** 2023-12-06

**Authors:** Mariela Subileau, Daniel Vittet

**Affiliations:** 1 University Grenoble Alpes, CEA, Inserm, IRIG, UA13 BGE, Grenoble, France

**Keywords:** lymphangiogenesis, lymphvasculogenesis, light sheet fluorescence microscopy, macrophage, ocular surface

## Abstract

**Purpose:**

Ocular lymphatic vessels play major physiological role in eye homeostasis and their dysfunction can contribute to the progression of several eye diseases. In this study, we characterized their spatiotemporal development and the cellular mechanisms occurring during their ontogenesis in the mouse eye.

**Methods:**

Whole mount immunofluorescent staining and imaging by standard or lightsheet fluorescence microscopy were performed on late embryonic and early postnatal eye mouse samples.

**Results:**

We observed that the ocular surface lymphatic vascular network develops at the early postnatal stages (between P0 and P5) from two nascent trunks arising at the nasal side on both sides of the nictitating membrane. These nascent vessels further branch and encircle the whole eye surface by sprouting lymphangiogenesis. In addition, we got evidence for the existence of a transient lymphvasculogenesis process generating lymphatic vessel fragments that will mostly formed the corneolimbal lymphatic vasculature which further connect to the conjunctival lymphatic network. Our results also support that CD206-positive macrophages can transdifferentiate and then integrate into the lymphatic neovessels.

**Conclusions:**

Several complementary cellular processes participate in the development of the lymphatic ocular surface vasculature. This knowledge paves the way for the design of new therapeutic strategies to interfere with ocular lymphatic vessel formation when needed.

Lymphatic vessels exert key roles for the maintenance of interstitial fluid homeostasis, the absorption of lipids and fat-soluble vitamins from the intestinal villi, and the transport of antigens and immune cell trafficking for adaptive immune responses.^1^ During the inflammatory processes, lymphatic vessels exert a resolutive role by their clearing properties, and impaired lymphatic function is associated with chronic inflammation.^2^^,^^3^ In addition to these well-known functions, some emerging novel organ-specific roles in tissue growth and repair have recently been identified that are related to lymphangiocrine signaling.^4^^–^^7^ Lymphatics then appear of particular importance in a large and growing number of physio-pathological situations. The mammalian ocular surface displays a rich lymphatic network comprising corneolimbal lymphatics connected with the underneath conjunctival lymphatic network covering the bulbar sclera.^8^^–^^10^ Ocular lymphatics have been reported to be critical in several eye pathological situations.^11^ Corneal lymphangiogenesis, the formation of new lymphatic vessels from pre-existing lymphatics of the limbus, is activated in inflammatory eye diseases.^12^^–^^14^ A beneficial role of lymphangiogenesis, because of its draining capacity, has also been postulated in cases of corneal edema.^15^ On the other hand, considering their role in immune surveillance, they can negatively interfere with corneal grafts by causing transplant rejection.^16^ In addition, ocular lymphatic vessels constitute a pathway for the metastasis of some ocular tumors.^16^ Finally, some studies have postulated the existence of a uveolymphatic pathway for aqueous humor drainage, which may have repercussions in the regulation of intraocular pressure.^8^^,^^10^ Although the presence of classical lymphatic vessels in the inner eye is controversial and the potential lymphatic involvement in aqueous humor drainage still debated,^17^ there is evidence for the involvement of a conjunctival aqueous humor outflow route after trabeculectomy is performed, and a recent work highlighted the existence of a direct lymphatic bridge between the Schlemm's canal and the limbal lymphatic vasculature.^18^ Thus a better understanding of ocular lymphatic vessel development seems to be essential for the elaboration of therapeutic strategies to modulate lymphatic vessel formation in ocular diseases.

In vertebrates, it is now admitted that lymphatic vasculature mostly originate from a venous endothelial cell progenitor subpopulation of the cardinal vein after the induction of the expression of the Prospero-related homeobox 1 (Prox-1) transcription factor.^19^ However, recent genetic lineage tracing studies have also confirmed and provided evidence for the existence of non-venous origins of the lymphatic vasculature. Such occurrence has been observed in the skin, the heart, and the mesentery, where the formation of lymphatics vessels was found to originate in part through lymphvasculogenesis, the formation and the assembly of lymphatic endothelial progenitors.^19^^,^^20^ Previous studies have pointed out that macrophages could participate in lymphatic vessel formation, at least in pathological situations, either by prolymphangiogenic cytokines secretion or by transdifferentiation into lymphatic endothelial cells.^21^ In the adult eye, macrophages have been demonstrated to contribute to corneal lymphangiogenesis in inflammatory conditions.^22^ Macrophages were also reported to contribute to the formation of lymphatic vessels leading to graft rejection during cornea transplantation.^23^ However, lineage tracing experiments designed to establish whether macrophages can transdifferentiate into lymphatic endothelial cells mainly showed that during mouse embryogenesis, lymphatics originate independently of the myeloid lineage.^24^ Despite this, the assumption that macrophages could transdifferentiate into lymphatic endothelial cells to contribute to lymphangiogenesis by integrating lymphatic neovessels cannot be totally excluded because another study reported the possibility that macrophages integrate into tumor lymphatic vessels.^25^ Besides, the existence of myeloid-derived lymphatic progenitor cells was assumed in several works aimed at deciphering the mechanisms of lymphatic vessel formation in adult mice in inflammation or tumor models or both.^26^

A recent study reported that the ocular surface lymphatic network organogenesis initiates at birth from the nasal side. The formation of a lymphatic trunk that further branches and encircles the entire ocular corneolimbal and conjunctival surface was described.^27^ However, the morphogenetic events involved in the expansion of the lymphatic network were not specified. In addition, a potential role of macrophages during the early developmental steps of ocular lymphatic vessels is not documented. The presence of both M1 and M2 macrophage subtypes was previously reported in the eyes of adult mice.^17^^,^^28^ In the present study, we further examine ocular surface lymphatic vessel development and analyze in detail the morphogenetic sequence of the events involved. We focused our interest on the spatiotemporal characterization of the ocular surface lymphatic vessel formation and in the potential contribution of macrophage transdifferentiation to lymphatic endothelial cells during this process.

## Material and Methods

### Mice and Eye Dissections

C57BL6/J wild type mice were used, in accordance with the statements of the ARVO (Association for Research in Vision and Ophthalmology) for the animal use guidelines in ophthalmology and vision research. The project was ethically approved by the French Ministry for Research and Education (agreement no. APAFIS 13689-2018022110161501 v2). Whole eyeballs from late embryos (E18.5), early postnatal neonates (P0 to P5), 11- and 30-day-old mice were collected by careful dissection, taking care to preserve the conjunctival and ocular surface tissues.^17^ They were fixed by overnight incubation with 4% paraformaldehyde in phosphate-buffered saline (PBS), washed, and stored at 4°C into PBS until further use. When required, and depending on the goal of the experiment, the anterior segments were dissected before further analysis.^17^

### Immunofluorescence Staining

Indirect immunofluorescence experiments were performed essentially as previously described.^17^ The samples were first incubated with a blocking and permeabilization solution (2% bovine serum albumin, 0.3% Triton X100 in PBS) overnight at 4°C. They were then incubated with primary antibodies overnight at 4°C. Goat anti-mouse LYVE-1 (AF2125), rat anti-mouse LYVE-1 (MAB2125), goat anti-mouse Prox-1 (AF2727), and goat anti-mouse CD80 (AF740) were obtained from Biotechne (Minneapolis, MN, USA). Rat anti-mouse CD31 (clone MEC13.3) was purchased from BD Biosciences (San José, CA, USA). Rabbit anti-mouse LYVE-1 (11-034) was from AngioBio (San Diego, CA, USA). Rabbit anti-mouse CD31 (ab124432) was obtained from Abcam (Cambridge, UK). Rat anti-mouse CD206 (MCA2235T) was from Bio-rad laboratories (Hercules, CA, USA). After several washes with PBS containing 0.3% Triton X100, the samples were incubated with secondary fluorescent antibodies for another overnight period at 4°C. Alexa fluor 488, Cyanin-3 or Cyanin-5-conjugated secondary antibodies displaying minimal cross-reactivity, all from Jackson Immunoresearch Laboratories (West Grove, PA, USA), were used. Finally, after several further washes with PBS containing 0.3% Triton X100, the samples were counterstained with Hoechst 33258 and postfixed with 4% paraformaldehyde.

### Fluorescence Imaging

Light sheet fluorescence microscopy (LSFM) imaging was performed using a light Sheet ZEISS Z1 microscope (Zeiss, Oberkochen, Germany) and ZEN Black software. The whole eyeballs were placed in the sample chamber either after embedding into 1% agarose or after fixation with superglue to a home-made sample holder. The LSFM images shown are two-dimensional reconstructions (maximum intensity projections) of series of acquired Z-stack images.

The imaging of whole mount immunostainings of the anterior eye were performed after cutting into four quadrants and flat mounting of the eye anterior segment with Fluorsave reagent (Merck Millipore, Darmstadt, Germany).^17^ Unless specified, all images are projections from z-stack acquisitions with a ZEISS AxioImager 2 fluorescence microscope equipped with an apotome and ZEN software.

## Results

### Characterization of the Developmental Kinetics of the Ocular Surface Lymphatic Vessel Network

**Figure 1.**
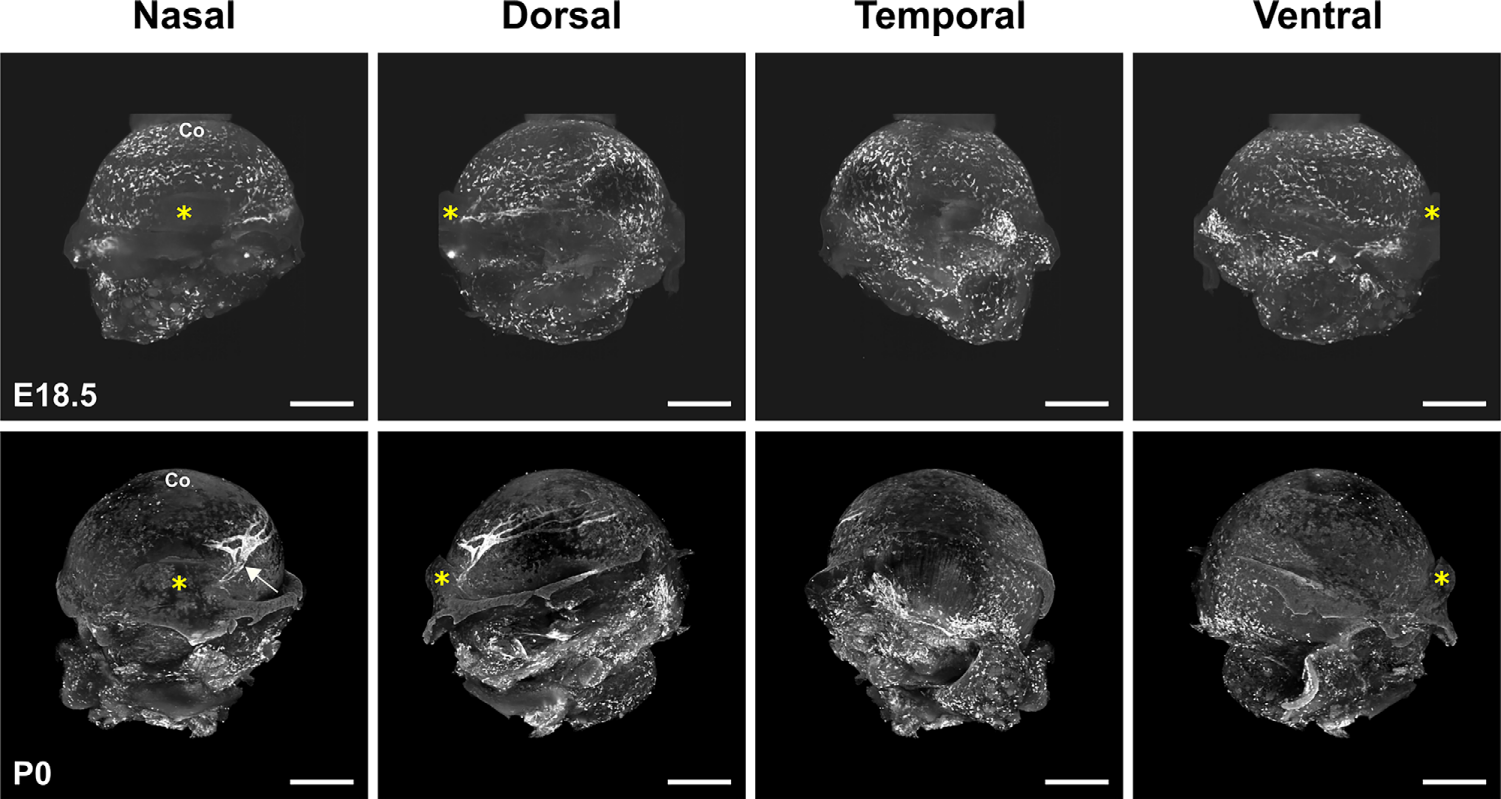
Initiation of ocular lymphatic development at birth. LSFM representative images of the LYVE-1 whole mount immunofluorescent staining of dissected right eyes of an E18.5 mouse embryo and of a P0 neonate. The multiview images corresponding to the projections at the four mentioned cardinal axes are shown. The *yellow asterisks* mark the position of the nictitating membrane at the nasal side of the eye. The *white arrow* points to the first nascent emerging lymphatic trunk. Note the presence of remaining connective and muscular tissues in the lower part of the ocular globe. Co, cornea. *Scale bars* for all panels: 500 µm. For LSFM representative images of LYVE-1 antibody negative control staining, see Supplementary Figure S1.

**Figure 2.**
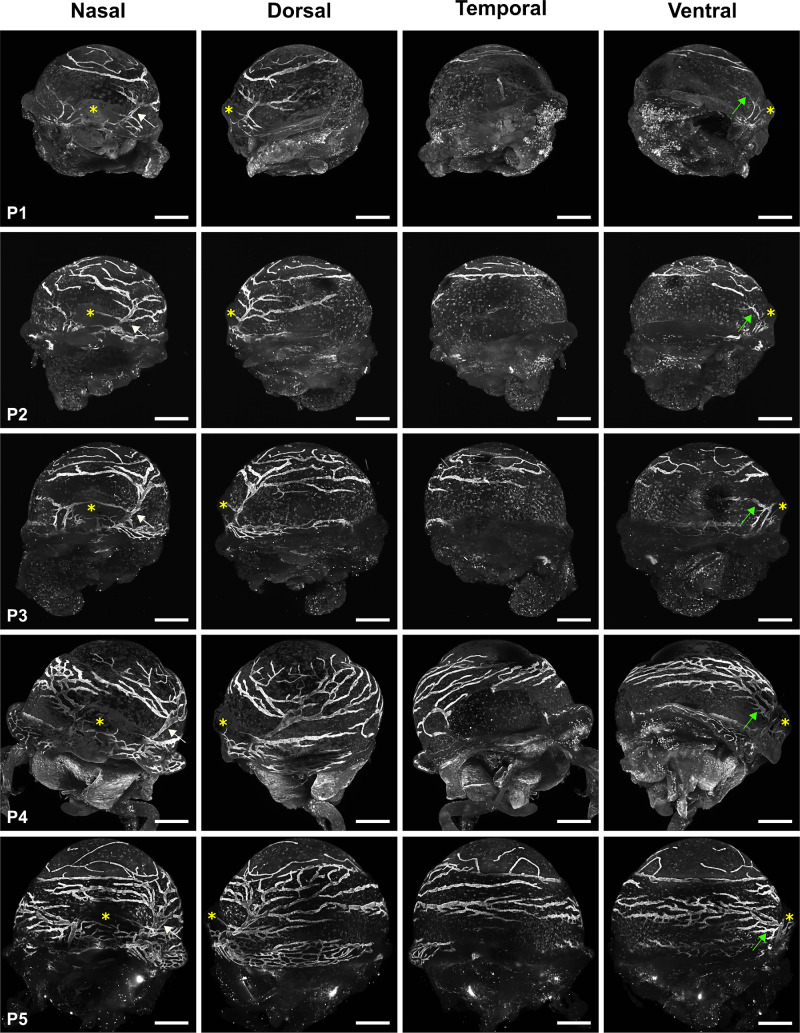
Expansion of the ocular surface lymphatic vessel network during early post-natal steps. Representative LSFM images of the multiview projections at the four cardinal axes of the LYVE-1 immunofluorescent staining of right eyes from P1 to P5. *Arrows* pointed to the roots of the lymphatic trunks which develops at both sides of the nictitating membrane marked by *yellow asterisks*. *White arrow*: root of the trunk from which the corneolimbal and the dorsal conjunctival lymphatic networks form; *Green arrow*: root of the trunk from which the ventral conjunctival lymphatic network forms. *Scale bars* for all panels: 500 µm. For LSFM representative images of LYVE-1 antibody negative control staining, see Supplementary Figure S1.

The spatiotemporal ocular surface lymphatic vessel formation was observed by LSFM imaging of the lymphatic vessel endothelial hyaluronan receptor 1 (LYVE-1) immunofluorescent staining. LYVE-1 represent a powerful antigenic marker to visualize the ocular surface lymphatic vasculature.^17^ The anti-LYVE-1 antibody used in LSFM imaging experiments has previously been shown to display high specific reactivity with minimal background, allowing the unambiguous visualization of LYVE-1–positive cells.^29^^–^^31^ Negative controls are illustrated in Supplementary Figure S1. The formation of ocular surface lymphatic vessels appears to initiate at birth (Fig. 1). At E18.5, a high density of single LYVE-1–positive cells are observed, which cover the whole surface of the eye, but no cord-like structures evoking lymphatic vessels could be found in most eyes at this stage. On some occasions, the first signs of LYVE-1–positive cell alignments are noticed (Supplementary Fig. S2). The emergence of a LYVE-1-positive nascent vessel evoking a large sac is observed around P0, adjacent to the dorsal side of the nictitating membrane. At this step, the corneolimbal blood vascular plexus has already developed, as assessed by CD31 pan-endothelial marker expression (Supplementary Fig. S3). This primary lymphatic vascular structure appeared to correspond to the root of a lymphatic initial trunk that will develop during subsequent early postnatal steps (Fig. 2). Indeed, this vessel expands, branches into two parts between P0 and P1 and further develop by encircling the corneolimbus between P2 and P3 to cover the whole ocular corneolimbal and conjunctival surfaces at around P4/P5. Beyond P1/P2, we noticed the formation of another lymphatic vessel primitive trunk on the ventral side of the nictitating membrane, which will expand thereafter. Then two different vessel roots, emerging on both sides of the nictitating membrane, are developing. The first one will form the dorsal conjunctival lymphatic vessel network and will contribute to the formation of corneolimbal vessels, whereas the second will form the ventral conjunctival lymphatic network. This is consistent with the observations in adults of two large lymphatic trunks on each side of the nictitating membrane, draining either the dorsal or the ventral part of the eye.^17^ Our observations are also in accordance with the polarized distribution of the conjunctival lymphatic network draining lymph at the nasal side, where the nictitating membrane is located, as previously reported in rodents and in human eyes.^17^^,^^27^^,^^32^

**Figure 3.**
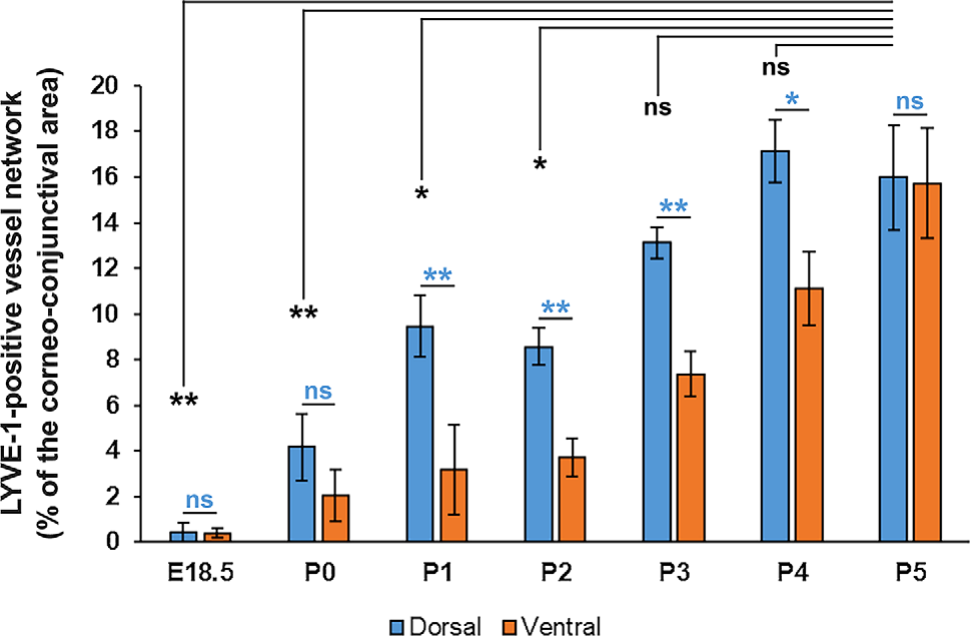
Quantitative analysis of the early steps of the ocular surface lymphatic vessel network formation. The measurements were performed on the images corresponding to the ventral and the dorsal lymphatic vessel network, respectively, facing the left and the right sides of the nictitating membrane in right eyes. The lymphatic vessel density was measured using ImageJ as the percentage of the corneal and of the conjunctival area that was occupied by LYVE-1–positive vessels. Single LYVE-1–positive cells were excluded from the analysis by prior image treatment. Data are the mean ± SEM of three (E18.5, P3, P4), four (P1, P2, P5), or seven (P0) different right eye samples. *Blue characters*: ** *P* < 0.01, * *P*< 0.05, dorsal value significantly different from corresponding ventral value; ns, not significant; using an unpaired Student's *t* test. *Black characters*: ** *P* < 0.01, * *P* < 0.05, dorsal value significantly different from P5 dorsal value; ns, not significant; using an unpaired Student's *t* test.

**Figure 4.**
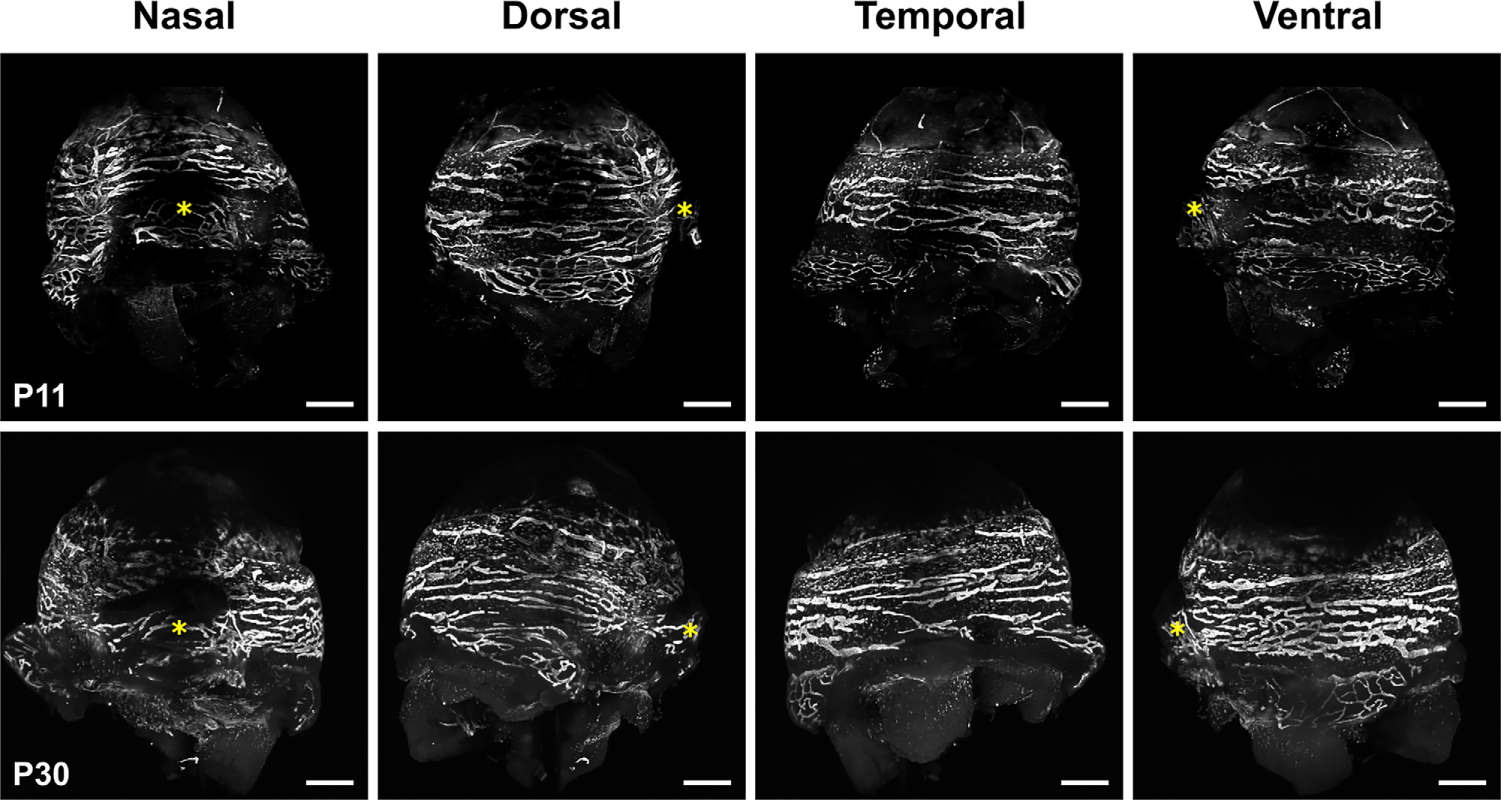
Views of the final maturation and remodeling of the ocular surface lymphatic vessel network. Representative LSFM images of the LYVE-1 immunofluorescent staining of left eyes at P11 and P30. The *yellow asterisks* mark the position of the nictitating membrane. *Scale bars* for all panels: 500 µm. For LSFM representative images of LYVE-1 antibody negative control staining, see Supplementary Figure S1.

The quantitative analysis confirms that the development of the LYVE-1-positive ocular surface lymphatic vessel network is achieved at early postnatal steps (from E18.5/P0 to P4/P5), and the delayed formation of the ventral network compared to the dorsal one (Fig. 3). Although a large part of the lymphatic network has developed by P4/P5, as assessed by the plateau values of the dorsal ocular lymphatic vessel area, the network still matures and remodels and appears completed at P30, exhibiting a similar organization and distribution pattern as observed in late adult stages (Fig. 4).^17^ Interestingly, the ocular lymphatic network seems to be functional early because lymphatic valves, whose formation are known to be induced by mechanosensing of the lymph flow,^33^^,^^34^ are detected at P3 (Supplementary Fig. S4). We can then expected a lymph circulation at the ocular surface at early developmental steps.

### The Morphogenetic Events Involved in Ocular Surface Lymphatic Vessel Formation

**Figure 5.**
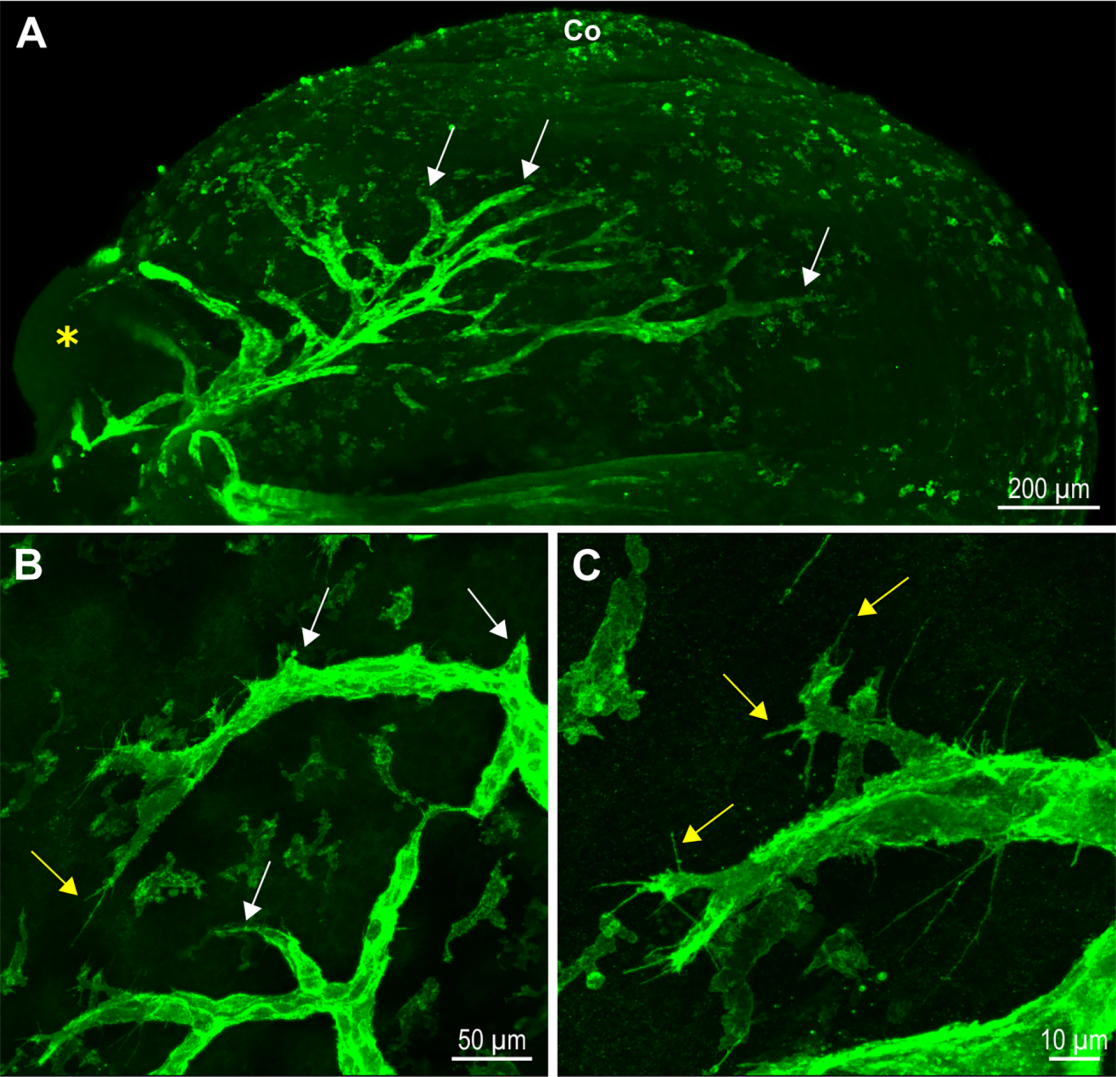
Sprouting lymphangiogenesis for ocular lymphatic vessel network expansion. (**A**) LSFM image of the sprouting LYVE-1-positive lymphatic vessel dorsal network of a right eye at P1. The *yellow asterisk* marks the nictitating membrane, and *white arrows* point to some sprout extremities. Co, cornea. (**B, C**) LYVE-1 immunofluorescent staining images of lymphatic sprouts observed at P1 after flat mountings of eye anterior segments. *White arrows* point to buds, and *yellow arrows* point to some filopodia.

Lymphangiogenesis, which corresponds to the sprouting and the formation of new vessels from preexisting ones, appears to constitute an important cellular mechanism involved in the expansion of the nascent LYVE-1–positive trunks that emerge adjacent to the nictitating membrane from P0 (Fig. 5A). Indeed, LYVE-1–positive sprouts exhibiting several buds are present at early developmental stages (Fig. 5B). Moreover, some LYVE-1–positive cells with several filopodia that sense their environment could be observed at the front of the lymphatic sprouts (Fig. 5C). Interestingly, in several cases, the cells at the extremity of the sprout, expressing Prox-1, appear to lack or to poorly express LYVE-1, suggesting that Prox-1 precedes LYVE-1 expression during lymphangiogenic sprouting (Supplementary Fig. S5).

**Figure 6.**
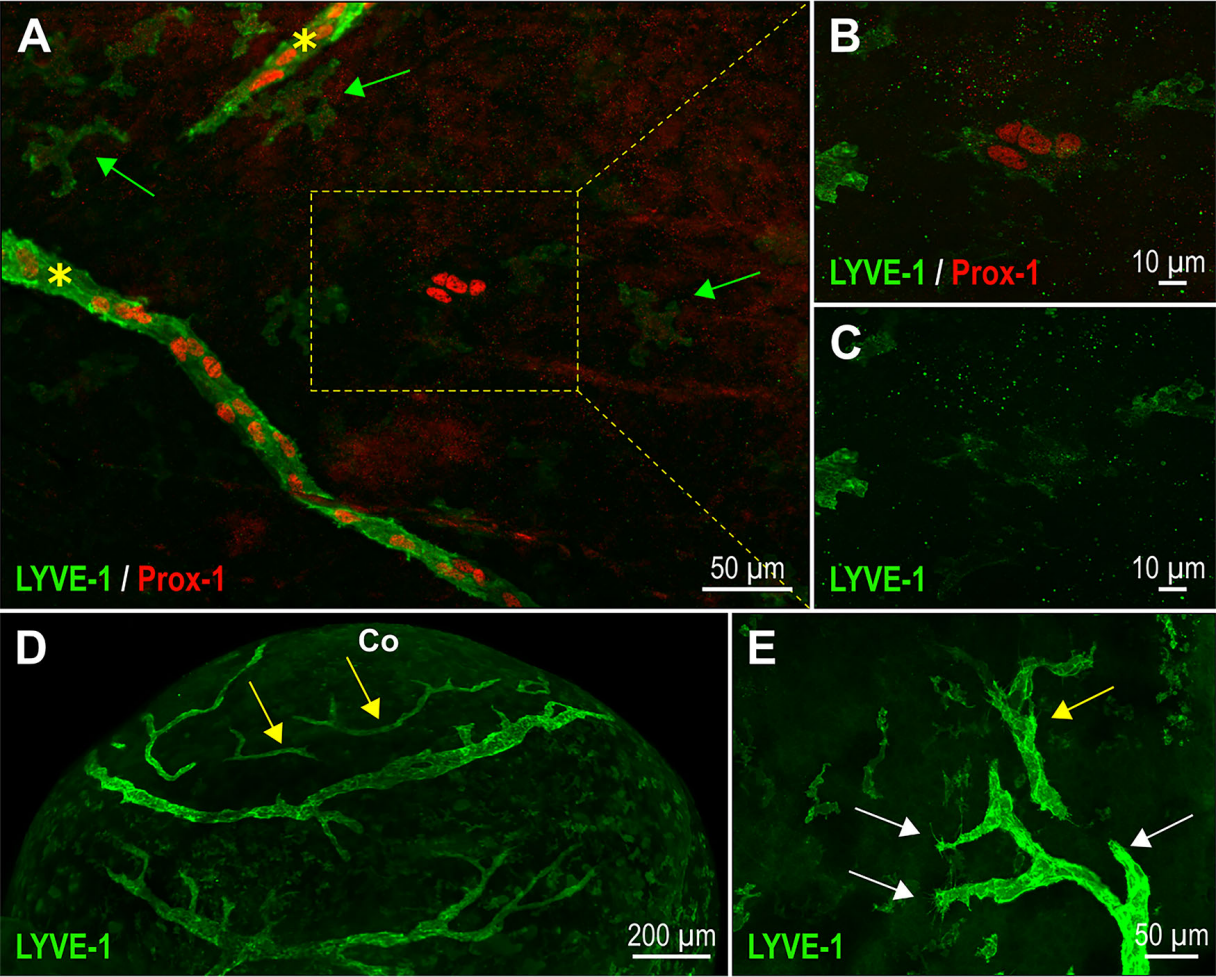
Lymphvasculogenesis constitute a cellular process for ocular lymphatic vessel development. (**A**) Visualization of the presence of a LYVE-1+ Prox-1+ cell cluster (*box*) at the P0 ocular surface after whole mount immunofluorescent staining of the eye anterior segment and flat-mounting. The *yellow asterisks* mark the location of two lymphatic sprouts. The *green arrows* point to some isolated LYVE-1–positive cells that correspond to macrophages. (**B**, **C**) Imaging of the boxed LYVE-1+ Prox-1+ lymphatic cell cluster at a higher magnification. (**D**) LSFM imaging of some LYVE-1-positive lymphatic vessel fragments (*yellow arrows*), which have formed at the corneal and corneolimbal surfaces of a P1 neonate. Co, cornea. (**E**) LYVE-1 immunofluorescence staining image obtained at P1, allowing the view of both lymphangiogenic sprouting (sprout extremities are pointed by *white arrows*), and a lymphatic vessel fragment, which could have been formed by lymphvasculogenesis (*yellow arrow*).

Although sprouting lymphangiogenesis appears to be a major process for corneolimbal and conjunctival lymphatic network expansion, a lymphvasculogenesis process could also be involved. One can observed the independent formation of LYVE-1–positive isolated cell clusters that develop cord-like structures. These cell clusters, which are also Prox-1 positive, which establishes their lymphatic identity, are mainly seen at the surface of the cornea above the corneolimbus. Such LYVE-1–positive and Prox-1-positive lymphatic cell clusters are illustrated in Figures 6A to 6C. Their formation has been routinely observed with the different pups during the time period ranging from P1 to P3. Cord-like structures evoking vessel segments were noticed thereafter (Fig. 6D), strongly suggesting that these lymphatic committed cell clusters may further develop, coalesce, or migrate to finally connect and assemble with the sprouting developing network at P4/P5. The initial non-connection of these lymphatic segments to the sprouting lymphatic vessel trunks strongly suggests that they originate from another cell source. Resident macrophages, suggested to contribute to the formation of new lymphatic vessels by secretion of lymphangiogenic factors for guidance purpose or by their incorporation into the neovessel and transdifferentiation, may be such a cell source.

### Evidence for Macrophage Contribution to the Ocular Surface Lymphatic Vessel Development

**Figure 7.**
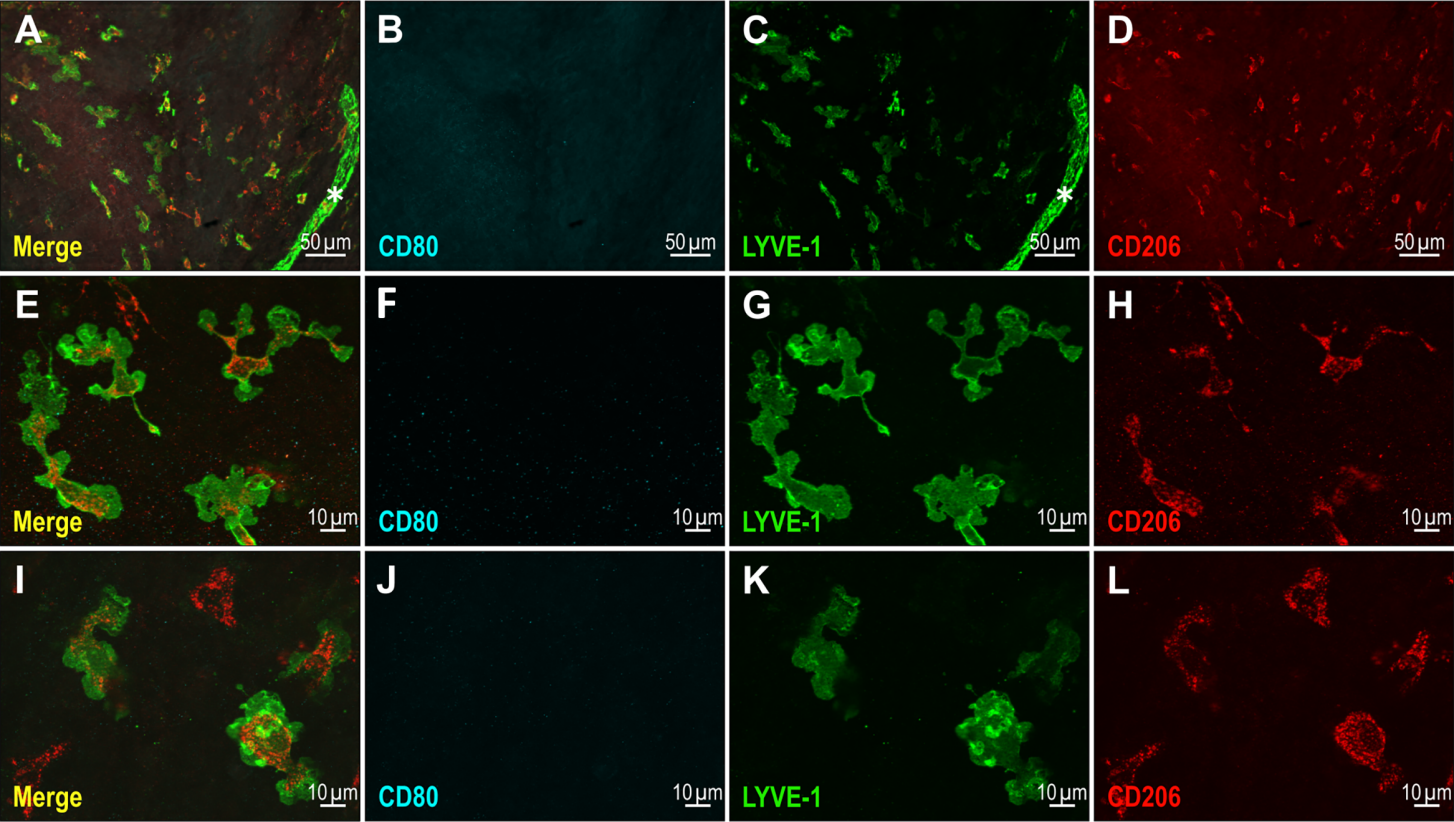
Ocular surface–scattered LYVE-1–positive cells display CD206 M2-macrophage antigenic marker expression. Illustrations of M1 (CD80) and M2 (CD206) macrophage subtype markers expressions in LYVE-1–positive cells after whole mount immunofluorescence staining and flat mounting of the anterior segments of the eyes at P1. The *asterisk* marks an LYVE-1–positive lymphatic vessel sprout.

Several macrophage subtypes expressed LYVE-1 at different expression levels.^35^^,^^36^ LYVE-1 expression has also been reported in the macrophages of murine adult eyes.^17^^,^^28^ In this study, we observed that dispersed LYVE-1–positive cells are present on the whole eyeball surface including the cornea, at the late embryonic stage (E18.5) (Fig. 1). A rich density of these cells is also seen at early postnatal stages at the time of the initial steps of the ocular lymphatic network development. Although they display heterogeneity in their LYVE-1 expression level and in their morphology, their macrophage identity was confirmed by CD11b expression, a pan-macrophage antigenic marker (Supplementary Fig. S6). Immunofluorescence studies of the eye anterior segment with specific M1 (CD80) and M2 (CD206) macrophage subtype markers, performed between P0 and P2, have revealed that the ocular surface LYVE-1–positive scattered cells, appeared to be predominantly representative of the regulatory M2 macrophage subtype at these developmental stages. Indeed, they express CD206 and are negative for CD80 (Fig. 7).^36^ Interestingly, CD80-positive M1 macrophages could be observed in adult eyes, suggesting further context-dependent polarization processes in the adult (Supplementary Fig. S7).

**Figure 8.**
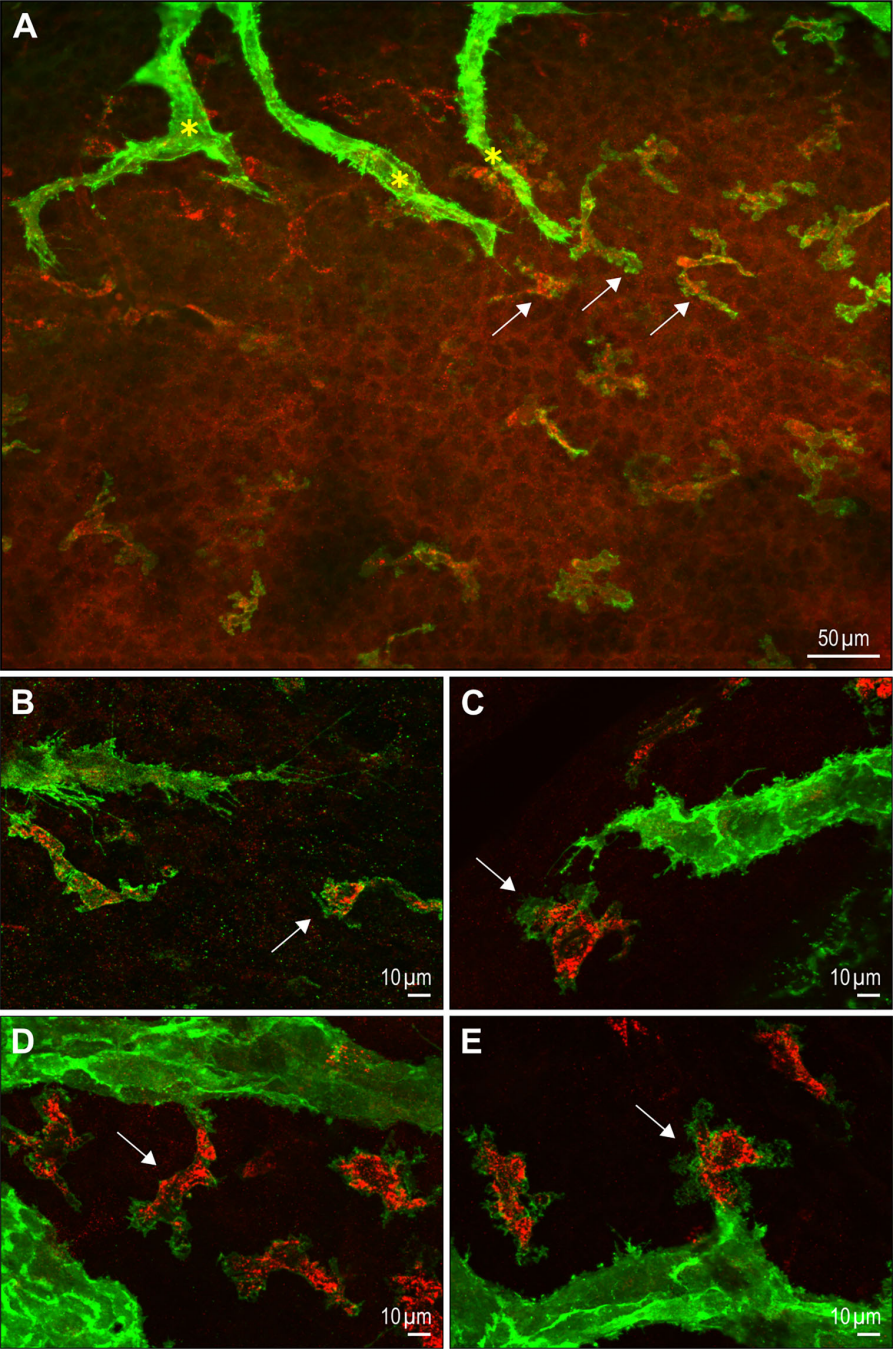
Morphological evidence for potential M2-macrophage contribution to ocular lymphatic vessel formation. Illustrations of immunofluorescent staining for LYVE-1 (*green*) and CD206 (*red*) at P1. (**A**) View of a corneolimbal surface part showing both lymphatic sprouts and single M2-macrophages. Note the high LYVE-1– and CD206-positive macrophage (*white arrows*) density and their close vicinity to the sprouts extremities marked by *yellow asterisks*. (**B–E**) Images evoking the association (**B, C**), the further attachment (**D**) and integration (**E**) into a developing lymphatic sprout, of an LYVE-1– and CD206-positive macrophage (*white arrow*) initially present in close proximity to a lymphatic sprout extremity.

The presence, at early developmental stages, of many single LYVE-1 and CD206-positive cells in close proximity with the sprouting lymphatic neovessels (Figs. 8A, 8B), is in favor of their further association and integration in the newly formed lymphatic vessels. On some occasions, it appears that these cells could potentially attach and connect to lymphatic endothelial cells to form part of the neovessel (Figs. 8C–8E).

**Figure 9.**
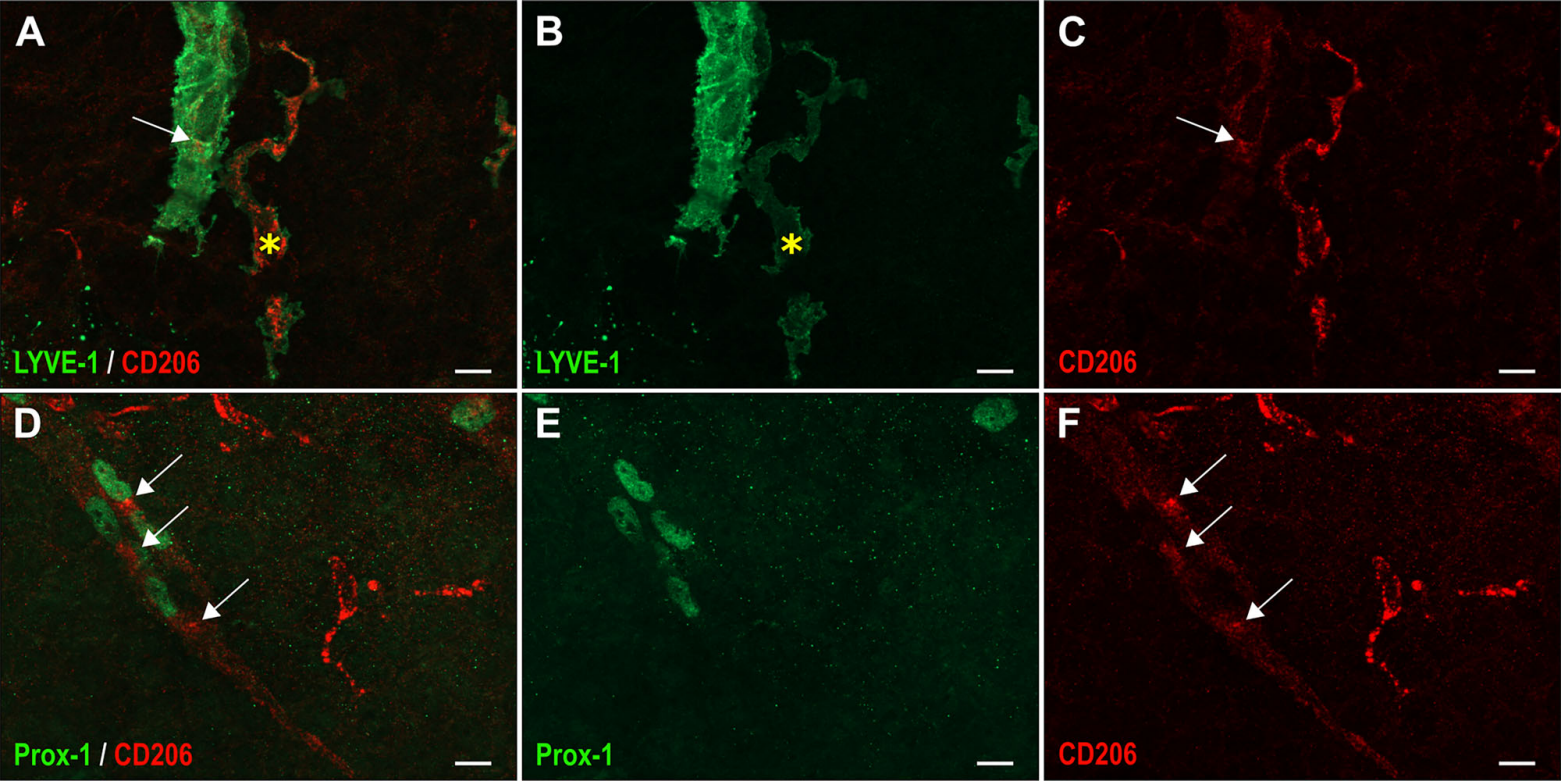
Evidence for the existence of mixed Prox-1– and CD206-positive cells in lymphatic neovessels. Whole mount double immunofluorescence staining with LYVE-1 and CD206 antibodies (**A–C**) or with Prox-1 and CD206 antibodies (**D–F**), of the developing ocular surface lymphatic vasculature at P1. The *yellow asterisk* marks a CD206-positive macrophage displaying a low LYVE-1 expression when compared to the LYVE-1 expression level of cells constituting the lymphatic neovessel. The *white arrows* point to the CD206 immunoreactivity in some LYVE-1– (*upper panels*) or Prox-1–positive cells (*lower panels*) of the lymphatic neovessel.

To know whether these macrophages can transdifferentiate into ocular lymphatic endothelial cells, we looked at the potential existence of cells expressing a mixed macrophage-lymphatic phenotype at P0/P1, during the early steps of lymphatic vessel formation. Some cells constituting the sprout were found to highly express LYVE-1 and concomitantly some remaining CD206 expression, whereas scattered macrophage at the vicinity display high CD206 and low LYVE-1 expressions (Figs. 9A–9C). Double Prox-1– and CD206-positive cells could also be observed, which was consistent with macrophage reprogramming into lymphatic endothelial cells once incorporated into lymphatic neovessels, because macrophages were not found to express Prox-1 at this developmental stage (Figs. 9D–9F).

## Discussion

In this study, we characterize the spatiotemporal and cellular mechanisms of development of the ocular surface lymphatic vascular system in the mouse. We confirmed that the timing of the ocular surface lymphatic vessel organogenesis occurs mostly during early postnatal steps, between P0 and P5, as previously described.^27^ We also confirm that the process initiates at the nasal side and that the final mature conjunctival lymphatic network is polarized from the nasal to the temporal side with a higher vessel density at the nasal side. The previous work of Wu et al.^27^ reported the initial formation of a lymphatic vessel at the nasal side that further extends by branching and encircling in both clockwise and counter-clockwise directions the whole surface of the ocular globe. Our results highlight substantial differences in the fact that we reported two different roots for the ocular surface lymphatic network, which develop on both sides of the nictitating membrane. They will drain the dorsal and ventral ocular region once the developmental process is completed. We also characterized that the lymphatic conjunctival network draining the dorsal part of the eye develops first, whereas the formation of the lymphatic ventral conjunctival network is slightly delayed. Moreover, the conjunctival dorsal network will in part be at the origin of the corneolimbal lymphatic vessels because it is the one that further develops by encircling the ocular globe and connects with lymphatic vessel segments that form at the surface of the cornea.

Actually, we provide novel insights in the characterization of the cellular mechanisms involved in the generation of the ocular surface lymphatic network. In addition to sprouting lymphangiogenesis, our results bring the evidence for the involvement of lymphvasculogenesis. This process appears to be mainly responsible for the formation of the corneolimbal lymphatic vascular plexus. Several lymphatic vessel segments arise at the surface of the cornea. They will migrate and connect with the conjunctival developing network and further encircle the ocular globe at the basis of the cornea. These two different morphogenetic cellular mechanisms are in accordance with the cellular mechanisms known to be involved in lymphatic vessel development in other mouse organs.^20^ Although we report on their involvement for lymphatic development in the mouse eye, they seem to generally apply to most organs. Such appearance of lymphatic vessel fragments at the ocular surface was already described in the adult mouse, but only during inflammatory pathological situations.^22^^,^^36^ Although a similar conjunctival lymphatic vessel polarization was postulated between mouse and humans eyes,^27^ the precise ocular surface lymphatic vessel distribution and the existence of similar morphogenetic events during ocular lymphatic vessel ontogenesis should be investigated in human eyes in future studies. Moreover, the respective relative contribution of the cellular mechanisms involved in the ocular lymphatic network formation should also be quantified.

We analyzed whether a third cellular mechanism for ocular lymphatic vessel formation could involve cells of the myeloid/macrophage lineage. Indeed, macrophages are key modulators of both angiogenesis and lymphangiogenesis processes either during tissue repair and remodeling or in pathological situations, including eye diseases. This is mainly achieved by the secretion of angioactive or lymphangioactive growth factors such as VEGF-A and FGF2^36^^,^^37^ or VEGF-C.^38^^–^^40^ These factors act in a paracrine manner, further attracting the neovessel tip cells facilitating the vessel network extension and stimulating blood and lymphatic growth. The M2-polarized macrophage subtype appeared to constitute the main contributor, displaying a higher potential than the other macrophage subsets, in the regulation of these processes.^37^^,^^41^^,^^42^ These M2-macrophage–supporting roles are well established in tumors^43^ and in some cases of functional tissue repair or development.^36^^,^^44^^,^^45^ During the ocular surface lymphatic vessel network ontogenesis, similar involvement of M2 macrophages seems to occur. Indeed, we observed a lack of proinflammatory M1 macrophages at early developmental stages. These M2 macrophages could be involved to link the lymphatic vessel segments derived from lymphvasculogenesis to the developing lymphatic network. Indeed, the observation of nonconnected lymphatic segments with tip cells displaying numerous filopodia are in accordance with an expansion process where filopodia sense the environment for guidance or to attract LYVE-1–positive macrophages. We could not exclude that these macrophages may provide a facilitating effect on lymphatic vessel formation by prolymphangiogenic factor secretion as described in previously published studies.^46^

Our results also provide some evidence that during early developmental processes, resident M2-like macrophage could contribute to the formation of new lymphatic vessel of the ocular surface by direct incorporation into the lymphatic neovessel. The question to know whether it corresponds to true macrophage transdifferentiation and/or to lymphatic endothelial mimicry awaits further investigation. Nevertheless, a lymphatic reprograming of macrophages does not appear before their physical integration into a lymphatic sprout. Indeed, in contrast to what was described by Maruyama et al.,^22^ during lymphangiogenesis in the inflamed cornea of mouse adult eyes we do not find macrophages expressing Prox-1, when nonintegrated into a lymphatic neovessel in formation. However, we cannot exclude the existence of a transient stage during which Prox-1 could be expressed, which may reflect that Prox-1 expression could be time and context dependent according to developmental stages or the existence of different origins for the myeloid/macrophage cells involved. Indeed, the heterogeneity in macrophage LYVE-1 expression levels could reflect a mixed macrophage cell population with different origins for their ontogeny or different stages of the differentiation from a same progenitor.^28^ It would then be interesting to know whether all LYVE-1–positive macrophage subpopulations display similar capacity to transdifferentiate into lymphatic endothelial cells and which signaling processes are involved during macrophage recruitment. Another important question would be to know whether the lymphatic endothelial cells from the different sources are functionally similar or if there may exist some differences leading to heterogeneity.

In conclusion, the ontogenesis of the ocular surface lymphatic vessel network formation appears to be more complex than previously reported. Our results indicate that the ocular surface lymphatic network develops by several different but complementary morphogenetic processes: sprouting from a lymphatic trunk originating behind the nictitating membrane but also differentiation of isolated lymphatic progenitors to develop lymphatic-committed cell clusters. Our results also support that macrophages can contribute by their direct integration in the expanding lymphatic vessels. Our results strongly suggest that M2-polarized tissue-resident LYVE-1–positive macrophages could transdifferentiate into lymphatic endothelial cells to constitute new lymphatic vessels. This is in accordance with the existence of myeloid-lymphatic progenitors which have been postulated to express M2-macrophage specific markers.^47^ Combined macrophage depletion and single cell RNA sequence studies of the expression profile of LYVE-1–positive cells at the initiation of the lymphatic network development would provide important information concerning the extent of macrophage contribution to lymphatic ontogenesis and about the different cell populations present during transdifferentiation. This information would allow the ability to selectively interfere with their potential involvement in the ocular lymphatic vessel developmental process.

## Supplementary Material

Supplement 1
