# Supplementary material for: Ontogenesis of the Mouse Ocular Surface Lymphatic Vascular Network
Source: Invest Ophthalmol Vis Sci. 2023 Dec 6;64(15):7. doi: 10.1167/iovs.64.15.7 (PMC10702784; doi:10.1167/iovs.64.15.7)
Supplement: Supplement 1 [file iovs-64-15-7_s001.pdf]

Supplementary Materials

Ontogenesis of the mouse ocular surface lymphatic vascular network.

Mariela Subileau & Daniel Vittet.

Supplementary Figures:

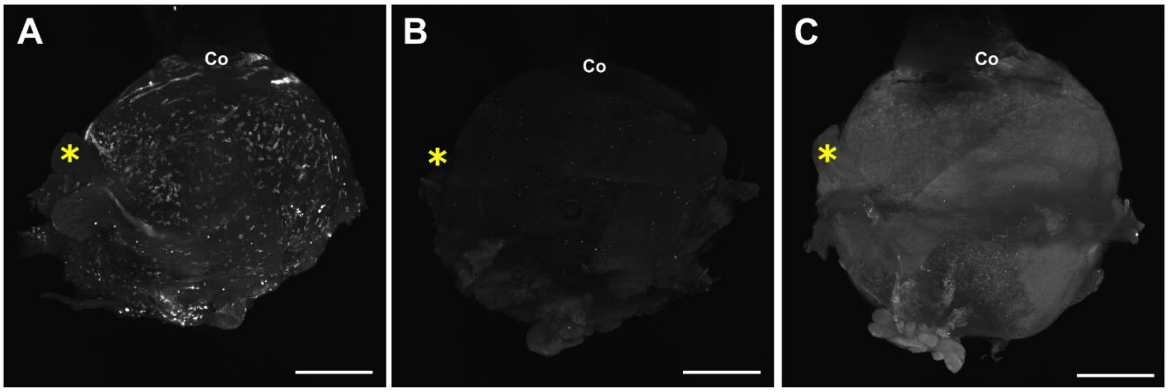

Fig. S1. Light Sheet Fluorescence imaging of LYVE-1 antibody negative controls after whole mount immunofluorescent staining of mouse eyes at P1. (A), staining with rat anti-mouse LYVE-1 antibody (MAB2125). (B), control staining in the absence of primary antibody. (C), control staining with irrelevant rat anti-Hemagglutinin proteins antibody (Ref: 11867423001; Roche Diagnostics, Mannheim, Germany). The image acquisitions were done under identical microscope settings and exposure times. The images were exported with strictly identical adjustments. The yellow asterisk marks the location of the nictitating membrane. Co, cornea. Scale bars for all panels: 500 µm.

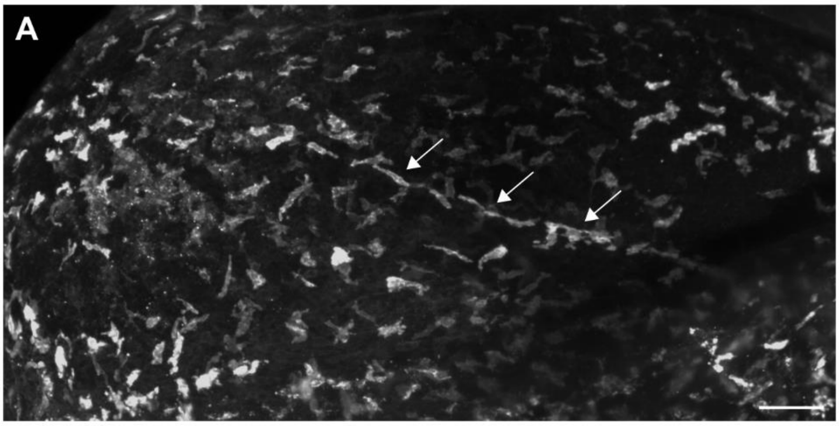

Fig. S2. First signs of lymphatic vessel morphogenesis in an E18.5 mouse embryo. LSFM imaging of LYVE-1 immunofluorescence staining of the ocular surface of an eye from an E18.5 embryo showing LYVE-1-positive cell alignments (white arrows) which may precede their assembly. Scale bar: 100 µm.

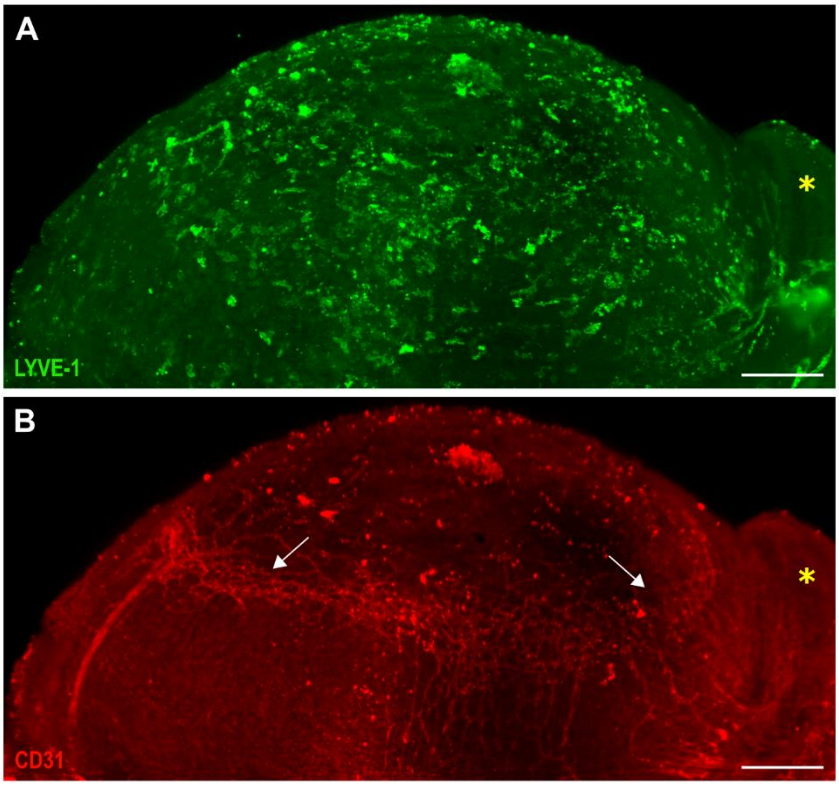

Fig. S3. The corneolimbal blood vascular plexus is already formed at the initiation of the lymphatic vessel network development. LSFM imaging of LYVE-1 (A) and CD31 (B) immunofluorescence stainings of the ocular surface of a left eye at P0. The arrows point to the primitive corneolimbal blood vascular plexus. The yellow asterisk marks the location of the nictitating membrane. One can note the lack of lymphatic LYVE-1-positive vascular cord-like structures at this developmental stage. Scale bars for both panels: 200 µm.

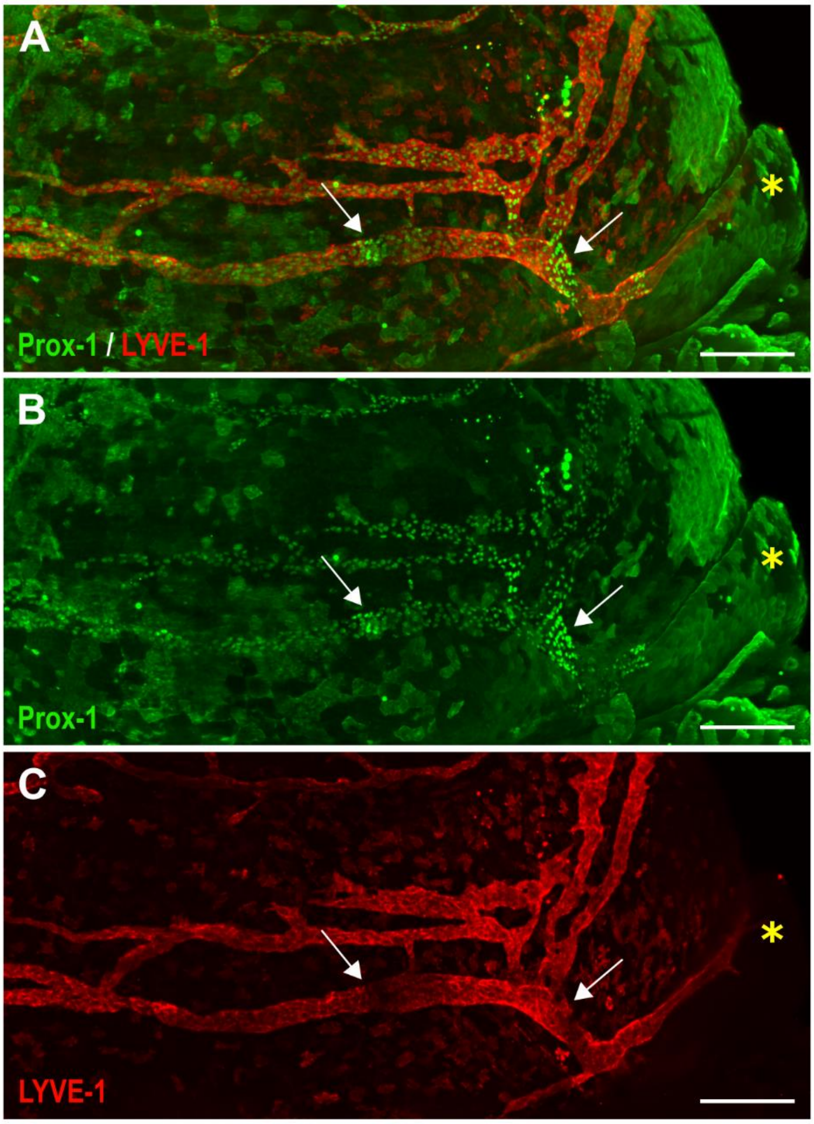

Fig. S4. Valves are present in lymphatic conjunctival vessels at P3. LSFM imaging of Prox-1 and LYVE-1 immunofluorescence stainings of a left eye at P3. The lymphatic trunk from which originates the dorsal conjunctival lymphatic network exhibits putative valves whose forming cells are LYVE-1-negative and are overexpressing Prox-1 (white arrows). The yellow asterisk marks the location of the nictitating membrane. Scale bars for all panels: 200 µm.

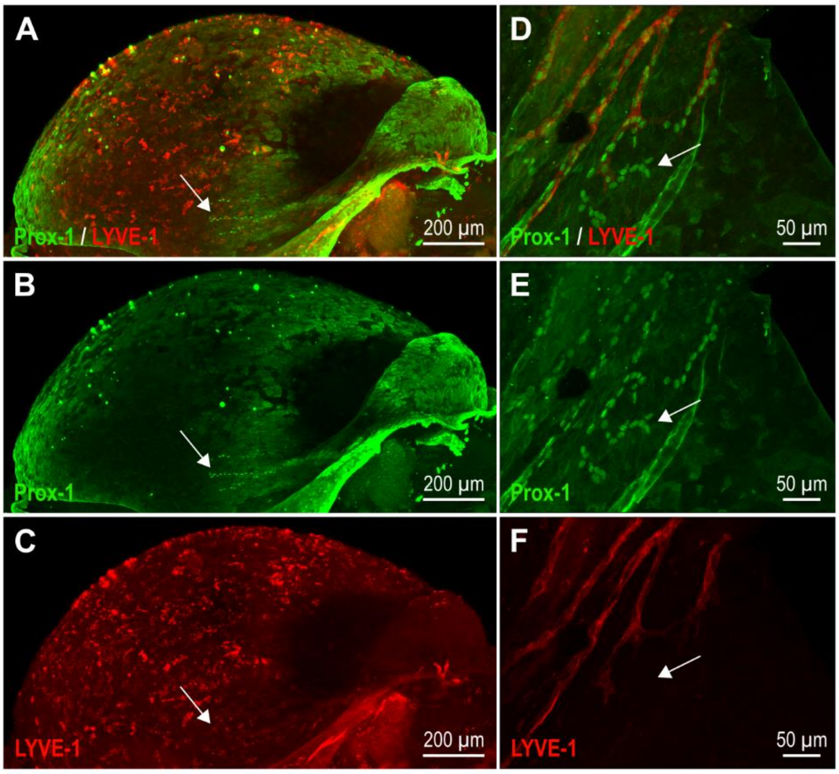

Fig. S5. Prox-1 expression marks the tip of forming sprouts during lymphatic vessel extension. (A to C), LSFM imaging at P0 of a Prox-1-positive nascent lymphatic vessel (white arrow) whose cells lack LYVE-1 immunoreactivity. (D to F), Prox-1 and LYVE-1 immunofluorescence staining images of lymphatic vessels observed on the surface of an eye anterior segment after flat mounting at P1. The white arrow points to the extremity of a Prox-1-positive sprout lacking LYVE-1 immunoreactivity.

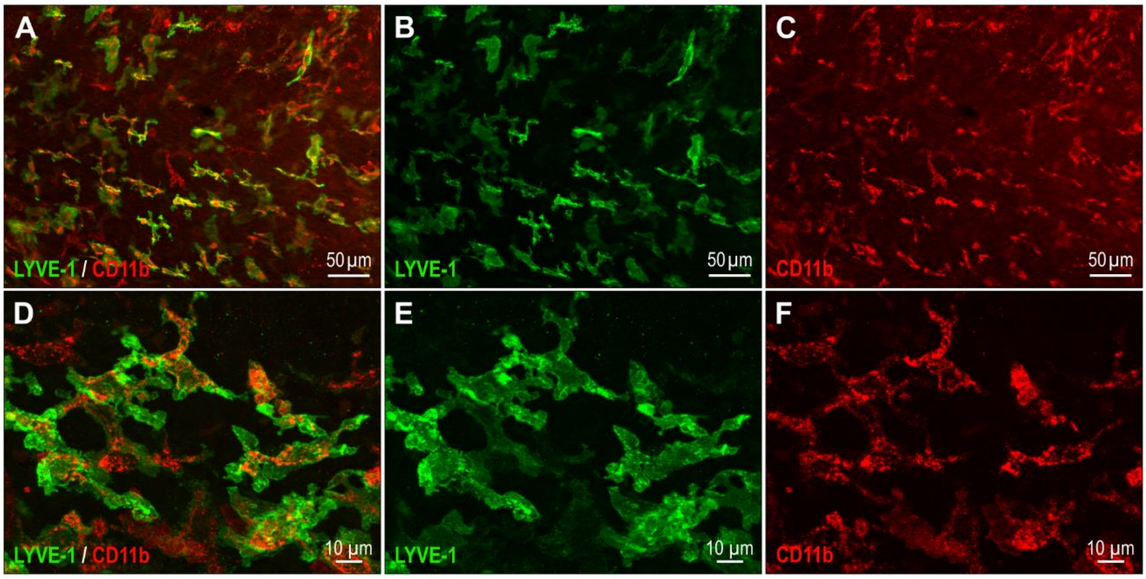

Fig. S6. Confirmation of the macrophage identity of ocular surface LYVE-1-positive single cells by CD11b co-expression. Representative views of whole mount double immunofluorescence stainings with LYVE-1 and CD11b antibodies of the corneolimbal region of the anterior segments of a mouse eye at P1.

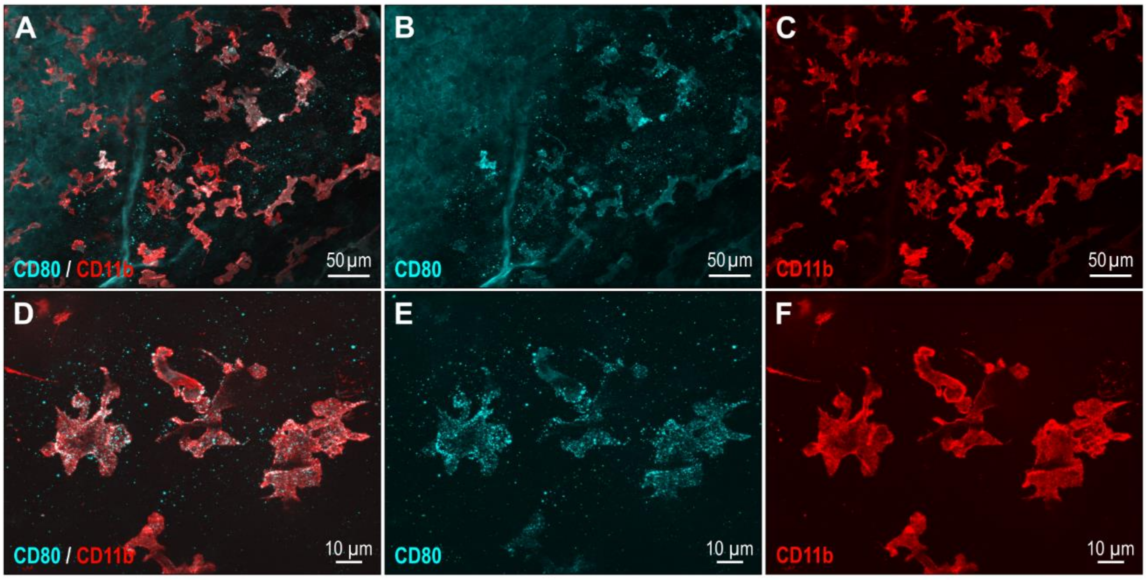

Fig. S7. CD80-positive M1-polarized macrophages can be observed in the corneolimbus of adult mouse eyes. Illustration of CD80 expression in some CD11b-positive macrophagic cells after whole mount immunofluorescence stainings and flat mounting of the anterior segments of mouse adult eyes. CD80-positive cells are mainly found at the interface between the cornea and the limbus.
